# The focus of temperature monitoring with zero-heat-flux technology (3M Bair-Hugger): a clinical study with patients undergoing craniotomy

**DOI:** 10.1007/s10877-018-0227-z

**Published:** 2018-11-22

**Authors:** Eero Pesonen, Marja Silvasti-Lundell, Tomi T. Niemi, Riku Kivisaari, Juha Hernesniemi, Marja-Tellervo Mäkinen

**Affiliations:** 1 Division of Anesthesiology, Department of Anesthesiology, Intensive Care and Pain Medicine, University of Helsinki and Helsinki University Hospital, Helsinki, Finland; 2 Department of Neurosurgery, University of Helsinki and Helsinki University Hospital, Helsinki, Finland

**Keywords:** Craniotomy, Body temperature, Zero-heat-flux temperature, Thermometer

## Abstract

In the noninvasive zero-heat-flux (ZHF) method, deep body temperature is brought to the skin surface when an insulated temperature probe with servo-controlled heating on the skin creates a region of ZHF from the core to the skin. The sensor of the commercial Bair-Hugger ZHF device is placed on the forehead. According to the manufacturer, the sensor reaches a depth of 1–2 cm below the skin. In this observational study, the anatomical focus of the Bair-Hugger ZHF sensor was assessed in pre- and postoperative CT or MRI images of 29 patients undergoing elective craniotomy. Assuming the 2-cm depth from the forehead skin surface, the temperature measurement point preoperatively reached the brain cortex in all except one patient. Assuming the 1-cm depth, the preoperative temperature measurement point did not reach the brain parenchyma in any of the patients and was at the cortical surface in two patients. Corresponding results were obtained postoperatively, although either sub-arachnoid fluid or air was observed in all CT/MRI images. Craniotomy did not have a detectable effect on the course of the ZHF temperatures. In Bland–Altman analysis, the agreement of ZHF temperature with the nasopharyngeal temperature was 0.11 (95% confidence interval − 0.54 to 0.75) °C and with the bladder temperature − 0.14 (− 0.81 to 0.52) °C. As conclusions, within the reported range of the Bair-Hugger ZHF measurement depth, the anatomical focus of the sensor cannot be determined. Craniotomy did not have a detectable effect on the course of the ZHF temperatures that showed good agreement with the nasopharyngeal and bladder temperatures.

## Introduction

Perioperative inadvertent hypothermia is common [1] and it increases blood losses [2], cardiovascular events [3] and wound infections [4] as well as impairs elimination of anesthetics [5]. Consequently, hypothermia may result in prolonged hospitalization [6] and increased mortality [7]. Postoperative shivering and feeling cold are uncomfortable for patients. Furthermore, malignant hyperthermia [8], fever or eventual therapeutic overheating, are conditions which demand immediate action. Accurate continuous perioperative core temperature measurement is thus indispensable in order to prevent or detect and treat these body temperature perturbations. Conventional reliable core body temperature measurements, e.g. pulmonary artery, esophagus, nasopharynx, or tympanum, are invasive, and less invasive methods are not suitable for continuous monitoring [9].

A noninvasive zero-heat-flux (ZHF) method, first demonstrated by Fox and Solman in 1970, was designed to measure deep body temperature superficially from the intact skin at sternum [10]. In this technique deep body temperature is brought to the skin surface as an insulated sensor with servo-controlled heating on the skin creates a region of ZHF from the core to the shell of the body. The temperatures of the skin and the deep tissue are assumed to be equal when heat flow between the skin and deep tissue ceases. The device was tested in awake subjects [11] and later during surgery in general anesthesia [12]. This technique, where a servo-controlled heating element is included, was developed further in Japan [13, 14]. These ZHF thermometers (Terumo) with reusable patient electrodes, placed on the sternum or forehead, have been clinically utilised [15, 16].

Recently, an improved commercial device (3M Bair-Hugger) applying the ZHF principle was introduced. In this system the response time is reduced to 3 min from the 10–20 min of the earlier appliances. The patient electrodes, positioned on the lateral forehead, are disposable. In an experimental evaluation of this system, ZHF temperatures measured on the surface of a manikin representing the human body and on a piece of bone cement, serving as a model of the frontal bone, were sufficiently accurate as compared with preset reference temperatures [17]. In clinical circumstances, the method has provided good agreement with conventional core temperature measurements in trauma, gynecological [18] and vascular surgery [19]. In cardiac surgery, however, the present ZHF method [19, 20] and another non-invasive temperature measurement technique via the forehead, with a double-sensor (Tcore) [21], seem to be less accurate. Recently, the Bair Hugger ZHF method has been reported to be reliable as compared with esophageal and iliac artery temperature measurements in surgical intensive care unit patients [22].

A key question is, what the sensor of ZHF as an indirect technique measures, i.e. which anatomical structures may be reached. The manufacturer of the Bair-Hugger ZHF device reports that the sensor reaches a depth of 1–2 cm below the skin surface [20]. Ideally, the focus of the Bair-Hugger ZHF sensor when placed on the forehead would reach the brain tissue for estimation of core body temperature. Preoperative MRI or CT scans of the head are available in adult patients undergoing elective craniotomy. In this observational study, we used elective craniotomy as a clinical model to assess the focus of the Bair-Hugger ZHF sensor in intraoperative core temperature measurement.

## Materials and patients

Ethical approval for this study (Ethics Committee 258/13/02/2014) was provided by the Ethics Committee for Surgery of the Hospital District of Helsinki and Uusimaa, Helsinki, Finland. All patients gave their written informed consent to participate in this study. We enrolled 30 adult patients scheduled for elective craniotomy.

### Anesthesia

The patients were premedicated with diazepam. Anesthesia was induced with thiopental sodium, fentanyl and rocuronium. Anesthesia was maintained either with intravenous propofol infusion (n = 15), or with 1 MAC isoflurane or sevoflurane in 50%/50% mixture with N_2_0 (n = 4), or with combination of propofol and isoflurane or sevoflurane (n = 10). In all patients, anesthesia was supplemented with remifentanil infusion and boluses of rocuronium. The target of arterial PCO_2_ was 4.0–4.5 kPa in mechanical ventilation.

### Temperature management

Before induction of anesthesia, a disposable ZHF temperature monitoring Bair-Hugger sensor (3M, St Paul, MN, USA) was positioned on the skin of the forehead at the mid orbital line above the eye brow contralateral to the site of the operation. After induction of anesthesia, a nasopharyngeal temperature probe (Mon-a-Therm General Purpose Temperature Probe 400TM, Covidien, Mansfield, MA, USA) at the depth of 10 cm from the nares [23], and a urinary bladder catheter (Mon-a-Therm Foley Catheter with Temperature Sensor 400TM, Covidien, Mansfield, MA, USA) were inserted. Patients were actively warmed during surgery using a forced air warming blanket (Bair Hugger Therapy, 3M, St Paul, MN, USA), an over-body conductive blanket (Hot Dog Augustine Temperature Management, Eden Prairie, MN, USA) or a warming mattress (Hot Dog Augustine Temperature Management, Eden Prairie, MN, USA). Additionally, warmed cotton blankets or space blankets were applied if needed. Operation room temperature was maintained at 19–20 °C.

### Temperature measurement

Body temperatures were recorded in an automated anesthesia system (Caresuite Anesthesia Manager 8.0, Picis Inc., Wakefield, MA, USA) and saved on-line in the hospital database. From the database, temperature measurements were retrieved at an interval of 5 min, which we considered long enough to detect a relevant change in body temperature between two consecutive time points. General anesthesia has a major impact on thermoregulation. Furthermore, nasopharyngeal and bladder temperature probes were placed after anesthesia induction. Thus, temperature measurements are reported starting from anesthesia induction. Craniotomy is associated with detachment of the brain from the cranium. Two separate time periods were analyzed: time period during the intact skull (i.e. before craniotomy) and time period after craniotomy.

### Radiological measurements

The manufacturer reports that the Bair-Hugger ZHF sensor reaches the depth of 1–2 cm. Consequently, placed on the forehead, the focus of the Bair-Hugger ZHF sensor was measured as the perpendicular distance from the skin surface to the point of both 1 cm and 2 cm to the intracranial direction in the vertical mid-orbital line. The same radiologist (RK, also neurosurgeon in addition to radiologist) conducted all measurements using the latest preoperative and the earliest postoperative head MRI or CT scans. In addition, the distance between the brain surface and the inner surface of the skull in the above defined line was measured as an estimate of postoperative intracranial air or fluid.

### Statistical analysis

Power analysis was not applicable in this observational study based on radiological imaging. The primary study objectives were twofold: to identify the intracranial focus of the Bair-Hugger ZHF sensor pre- and postoperatively; and to observe if there was a discontinuation time phase of the Bair-Hugger ZHF sensor temperature measurement at the time of craniotomy. The secondary objective was to measure agreement between the Bair-Hugger ZHF sensor temperature and the nasopharyngeal and urinary bladder temperatures.

MedCalc® statistical software (MedCalc Software, Ostend, Belgium) and SPSS 25 for Windows (IBM Corp, Armonk, New York, USA) were used for statistical analyses. Non-parametric approach was used due to small patient number. Friedman’s test was used for testing differences as a function of time. Spearman’s test was used for bivariate correlations. The agreement between two methods was assessed using the Bland–Altman random-effects approach for data of repeated measures. In addition, the percentage of measurement differences within the range of ± 0.5 °C was counted. p values < 0.05 were considered statistically significant. Data are expressed as median and inter quartile range (IQR) and, when appropriate, additionally as range (minimum and maximum). However, to account the effect of the whole patient population, the mean is used as a characteristic in the figure illustrating temperature changes as a function of time.

## Results

### Patient and operation data

Patient characteristics and operative data are presented in the Table 1. One patient was deleted due to failure in retrieval of temperature data. Otherwise, there were 19 females and 10 males.

**Table 1:** Demographic and operative data

Number of patients	29
Age (year)	59 (44–67)
Male/female	10/19
BMI	26.6 (23.9–31.6)
Duration of anesthesia (min)	205 (166–253)
Duration of surgery (min)	176 (125–210)
Position of the patient	
Supine	19
Lateral	7
Sitting	3
Surgery indication	
Meningioma	10
Glioma	7
Hemangioblastoma	1
Medulloblastoma	1
Arterial aneurysm	3
Trigeminal neuralgia	2
Moya-moya	2
Rhabdomyosarcoma metastasis	1
Breast cancer metastasis	1
Melanoma metastasis	1

### The focus of ZHF measurement preoperatively

A preoperative head CT scan was available in 12 patients and an MRI scan in 17 patients. The image was taken 33 (10–91) days before the operation. Assuming that ZHF reached the depth of 20 mm (the maximum depth reported by the manufacturer) from the forehead skin surface, the temperature measurement point (see the methods for the definition) reached the brain cortex in all except one patient. In the latter patient, there was a sub-arachnoid fluid mantel of 4 mm and the temperature measurement point was located in the fluid mantel. In all patients, the median measurement point was 4 (3–7) mm beneath the surface of the cortex. In 14 patients, the measurement point was at least 5 mm beneath the cortex surface. Assuming that ZHF reached the depth of only 10 mm (the minimum depth reported by the manufacturer) from the forehead skin surface, the temperature measurement did not reach the brain parenchyma in any of the patients and was at the cortical surface in two patients. In 11 patients, there was a sub-arachnoid fluid mantel ranging from 1 to 4 mm in thickness. There was an inverse correlation (R = − 0.506, p = 0.005) between the fluid mantel thickness and the temperature measurement point focus. In other words, less amount of sub-arachnoid fluid was associated with deeper intraparenchymal measurement point. There was no intracranial air in any patient preoperatively.

### The focus of ZHF measurement postoperatively

**Fig. 1:**
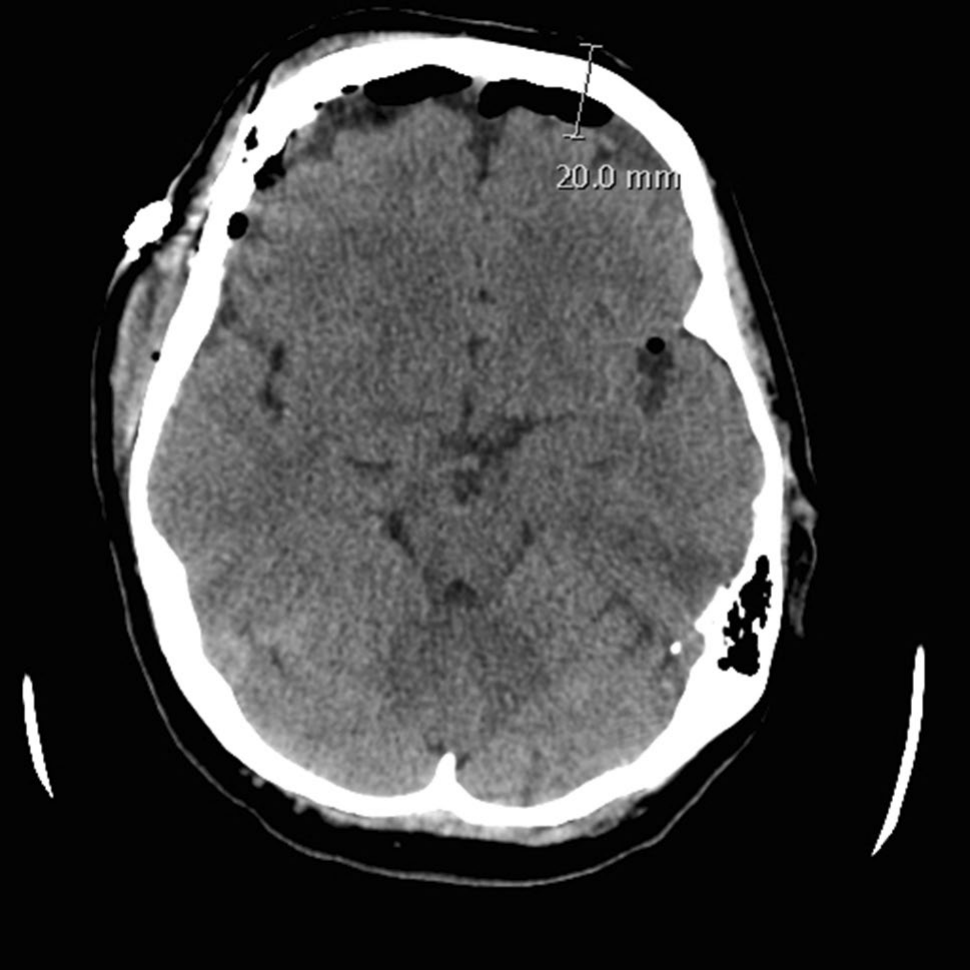
A representative postoperative axial CT scan. Distance from the skin to the cortical surface measured from the ocular papillary line 2 cm from the eyebrow

**Fig. 2:**
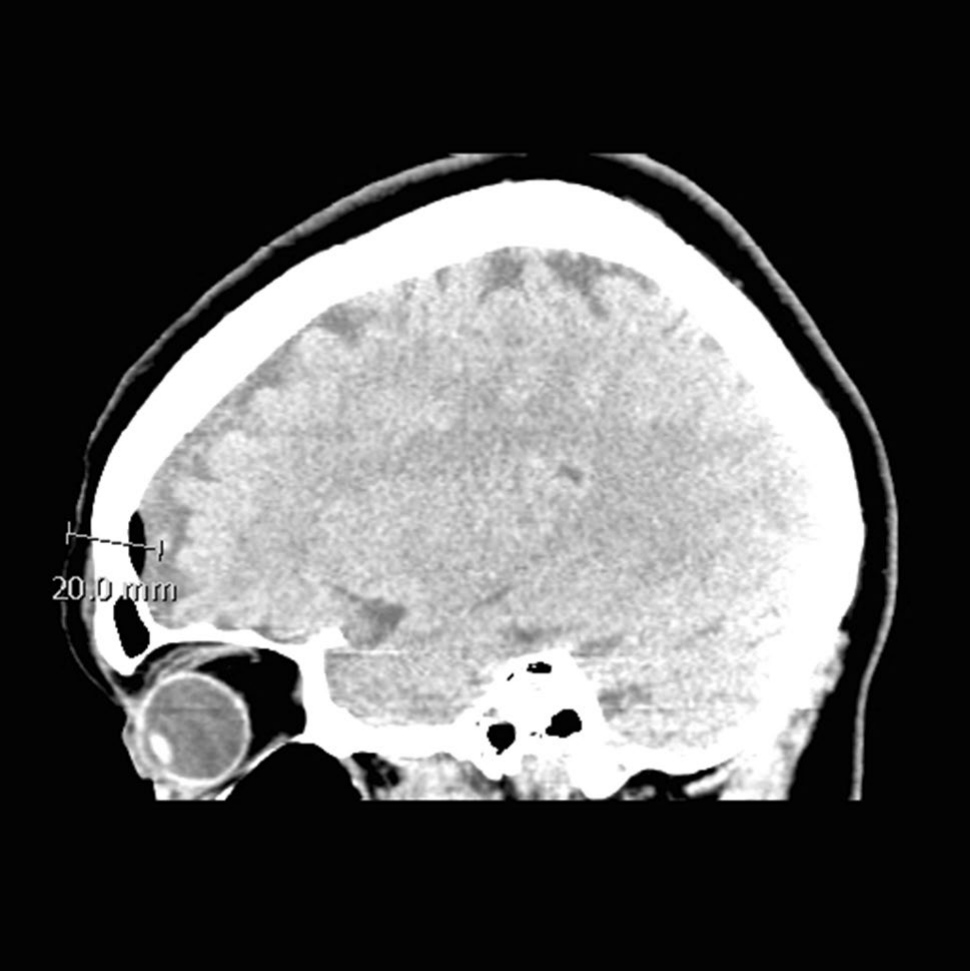
A representative postoperative sagittal CT scan. Distance from the skin to the cortical surface measured from the ocular papillary line 2 cm from the eyebrow

A postoperative image was available in 23 patients (13 CT and 10 MRI scans) in a median (IQR) of 1 (1–2) days postoperatively, ranging from 0 to 12 postoperative days. Representative postoperative axial and sagittal CT scans of the same patient are presented in the Figs. 1 and 2, respectively. Assuming that ZHF reached the depth of 20 mm from the forehead skin surface, the temperature measurement point reached the brain cortex in all patients. The median measurement point (IQR) was 4 (3–7) mm beneath the surface of the cortex, ranging from 0 to 10 mm. Assuming that ZHF reached the depth of only 10 mm from the forehead skin surface, the temperature measurement did not reach the brain parenchyma in any of the patients and was at the cortical surface in only one patient. Either a sub-arachnoid air (16 patients) or fluid (seven patients) but not both was observed in all postoperative images. In 13 patients, the air mantel was less than 1 mm thick and in the rest three patients, the thickness was 1, 6 and 8 mm in each. In another seven patients, a postoperative sub-arachnoid fluid mantel ranging from 1 to 8 mm was observed. Again, there was an inverse correlation (R = − 0.557, p = 0.006) between the air/fluid mantel thickness and the depth of the intraparenchymal temperature measurement point.

### Temperature measurements as a function of time

**Fig. 3:**
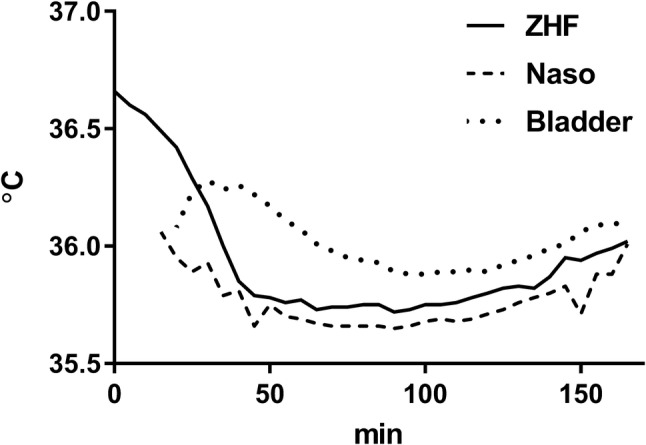
The zero-heat flux (ZHF), nasopharyngeal (Naso) and urinary bladder (Bladder) temperatures at 5-min intervals starting from anesthesia induction

After anesthesia induction, mean ZHF temperature was 36.7 ± 0.4 °C (Fig. 3). The first obtained nasopharyngeal temperature 15 min after anesthesia induction was 36.1 ± 0.5 °C and concomitant ZHF temperature 36.5 ± 0.4 °C. The ZHF temperature gradually declined and reached the nasopharyngeal temperature approximately 45 min after anesthesia induction. Thereafter, the ZHF and nasopharyngeal temperatures paralleled closely each other till the end of the operation so that ZHF temperature was approximately 0.1 °C higher than nasopharyngeal temperature (Fig. 3).

The time period consisting of both the preceding and following 30 min (i.e. 60 min together) from craniotomy was used to assess the effect of craniotomy on ZHF temperature measurement. During this period, there was no trend in ZHF temperatures (Friedman’s test) or change in pairwise comparison (Wilcoxon test) between any two consecutive time points (data not shown). We further calculated absolute differences in ZHF temperatures between all pairs of two consecutive time points. Similar to absolute ZHF temperature values, there was no trend in the calculated differences or change in pairwise comparison between any two consecutive time points (data not shown).

### The agreement between different methods of temperature measurement

**Fig. 4:**
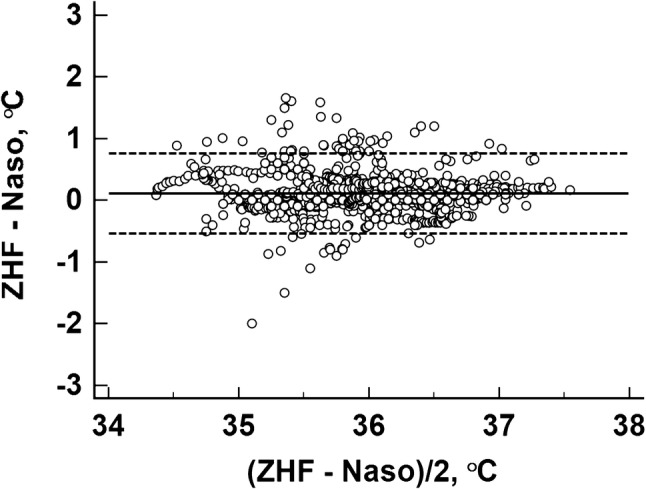
Bland–Altman plot between the zero-heat flux (ZHF) and nasopharyngeal (Naso) temperatures

**Fig. 5:**
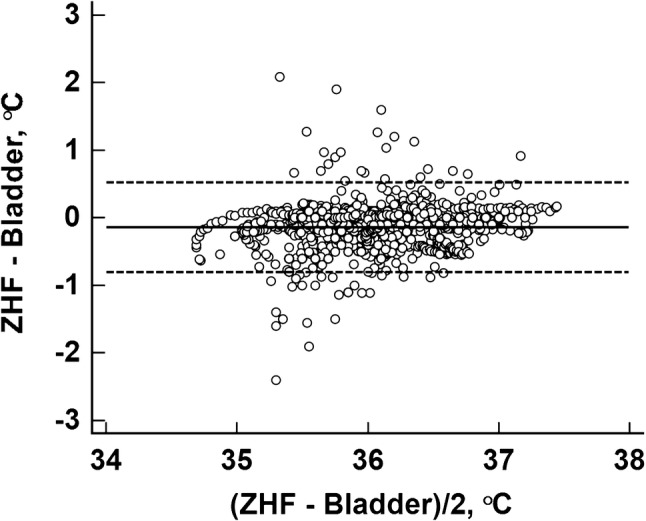
Bland–Altman plot between the zero-heat flux (ZHF) and urinary bladder (Bladder) temperatures

A priori, intention was to divide the study period to separate time phases before and after craniotomy. While we did not detect any effect of craniotomy on ZHF measurements, such division turned out to be irrelevant and agreement was tested during the entire study period. The agreement of ZHF temperature with the nasopharyngeal temperature was 0.11 (− 0.54 to 0.75) °C (Fig. 4) and 90.5% of the temperature differences were within 0.5 °C. The agreement of ZHF temperature with the bladder temperature was − 0.14 (− 0.81 to 0.52) °C (Fig. 5) and 88.5% of the temperature differences were within 0.5 °C.

## Discussion

Unlike conventional means of core temperature measurement, the ZHF method is indirect by nature. The temperatures of the skin and the deep tissue are assumed to be equal when heat flow between these two locations ceases. The forehead is an optimal place for a ZHF sensor, because there is practically no subcutaneous tissue even in an obese patient. For a surrogate of core temperature, it might be ideal, if the measurement range of the ZHF probe reached the brain cortex parenchyma. According to the manufacturer, the Bair-Hugger ZHF sensor reaches the depth of 10–20 mm. Assuming the depth of 20 mm, the temperature measurement point was in the brain cortex in all except one of our patients in preoperative images and in all patients in the postoperative images. On the other hand, if the depth of only 10 mm is assumed, the cortex surface was barely reached only in one patient postoperatively but in none of the patients preoperatively. While either no sub-arachnoid fluid or only of 1 mm was observed in all except three patients preoperatively, sub-arachnoid fluid was not a major confounding factor in the preoperative results of the present study.

As conductance of heat in water is good, one could speculate that subarachnoid fluid probably does not impair the performance the of the ZHF method. Air, on the other hand, conducts heat poorly. Craniotomy is potentially associated with detachment of the brain from the cranium. As a surprise we did not observe any effect of craniotomy on the course of the temperatures obtained by the ZHF sensor. Although we did not measure intracranial air intraoperatively, its’ occurrence during the operation is likely, because air was found in most of the patients in the postoperative image. It might be assumed that any intraoperative air layer was immediately warmed up by convection of the circulating blood in the adjacent brain and cranium. Consequently, the agreement between the ZHF probe and nasopharyngeal temperatures was 0.11 ± 0.33 °C, i.e. good. A difference less than 0.5 °C between two temperature measurement methods is traditionally accepted as a criterion of good agreement [24]. In this study, 90.5% of the differences between the ZHF probe and nasopharyngeal temperatures were within this criterion.

The ZHF forehead temperature before anesthesia induction was 36.6 ± 0.6 °C. Using proton magnetic resonance spectroscopy, the frontal lobe temperature approximately of the same range (37.2 ± 0.6 °C) has been measured non-invasively in awake healthy adult volunteers [25]. After placement of the nasopharyngeal probe under general anesthesia, the ZHF temperature was 0.4 °C higher than the concomitant nasopharyngeal temperature. The ZHF temperature declined steadily, approaching the level of the nasopharyngeal temperature within 45 min after anesthesia induction. Thereafter, these two temperatures paralleled closely each other. The decrease in the ZHF temperature of 0.8 °C after anesthesia induction may be partly due to cooling effect of the preparations before surgery. However, we believe that mostly the ZHF temperature was reduced due to general anesthesia that induces heat redistribution between different compartments of the body [26]. Indeed, we have previously observed a similar pattern of the ZHF temperature at the beginning of general anesthesia in patients undergoing vascular surgery [19]. Nasopharyngeal temperature measurement is generally used as a surrogate for brain temperature in craniotomies [27].

The optimal core temperature in craniotomy is not known. Despite active warming, our patients cooled down to the temperature of 35.7 °C. At the end of the operation the core temperature increased to over 36 °C. Both hypo- and hyperthermia are harmful to the brain. In patients suffering from spontaneous intracerebral hemorrhage, increased brain temperature resulted in spreading depolarizations in the cortex [28]. Likewise during rewarming in cardiac surgery, overheating of the patients impaired postoperative cognitive function [29]. On the other hand, increased surgical bleeding tendency due to unintended hypothermia may be detrimental in the brain [2]. Still, therapeutic hypothermia has been reported to improve outcome of patients undergoing craniotomy for intracranial hematomas or severe traumatic brain injuries [30].

As a major limitation of this study, the time interval between the preoperative images and surgery was considerably long in some of our patients. Furthermore, one should be cautious when generalizing the present results to patients without intracranial pathology, although preoperative sub-arachnoid fluid seemed not to have major confounding effect. While a patient population with available brain images are hard to find, this is the first study estimating the measurement focus of the ZHF probe on the forehead. As a strength, automated continuous recording of temperature measurements made a dynamic approach possible to investigate the interrelationship of different temperature measurement methods as a function of time. An interval as long as 5 min between two consecutive measurements ensured that, instead of repeating same measurements within a short time interval, true changes of body temperatures were detected.

In respect to the anatomical dimensions beneath the skull, the manufacturer reports a fairly large range of the measurement depth for the Bair-Hugger ZHF sensor. Within this range, the focus of the sensor may reach the brain cortex either in all the patients, none of the patients or anything in between. Intuitively thought, it might be ideal to reach the brain parenchyma for the estimation of the core body temperature. That cannot be either concluded or excluded according to the present data and the reported measurement depth. Still, good agreement of the Bair-Hugger ZHF system with other means of core temperature measurement has been obtained in most clinical studies published thus far [18–20, 22, 31].
